# Assessing the impact of discontinuation of formulary prior authorization on antibiotic prescribing

**DOI:** 10.1017/ash.2024.92

**Published:** 2024-09-25

**Authors:** Teagan Zeggil, Tony Nickonchuk, Elissa Rennert-May, Irina Rajakumar

**Affiliations:** 1 Alberta Health Services, Edmonton, AB, Canada; 2 Drug Utilization and Stewardship Pharmacist, Alberta Health Services, Edmonton, AB, Canada; 3 Division of Infectious Diseases, Department of Medicine, Foothills Medical Center, Calgary, AB, Canada; 4 Pharmacy Clinical Practice Leader, Alberta Health Services, Calgary, AB, Canada

## Abstract

**Objective:**

To compare prescribing patterns of restricted antimicrobials before and after the removal of prior authorization and to develop a prospective audit and feedback program to mitigate the potential inappropriate prescribing of these antimicrobials.

**Methods:**

An interrupted time-series analysis assessing the trends in antibiotic use was conducted between May 2020 and February 2023 in large urban hospitals, where all ASP activities were discontinued in May 2022 and a pilot prospective audit and feedback (PAF) program was initiated in January 2023.

**Results:**

The collective change in restricted antibiotic utilization after the removal of prior authorization was trending towards increased utilization but was not statistically significant. With the PAF program, 9.8% of patients were identified by the antimicrobial stewardship pharmacists as requiring intervention. Within these patients, 19 different recommendations were made, with the most common being to narrow the therapeutic spectrum (47.4%). Stewardship interventions suggestions were accepted (full and partial) 69.2% of the time.

**Conclusions:**

Although there were some small statistically significant changes detected for a few antibiotics, there were no situations where those changes remained significant after appropriate controls were added to the analyses. As such, the intervention may not have had any statistically significant impact on DDDs of the studied antibiotics.

## Introduction

The Infectious Disease Society of America (IDSA) guidelines recommend prospective audit and feedback (PAF) and prior authorization as core components of an antimicrobial stewardship program.^1,2^ Prior to May 2022, prior authorization and PAF were frequently utilized as antimicrobial stewardship strategies in Calgary to optimize antimicrobial prescribing. Due to the implementation of a new electronic medical record and provincial standardization, prior authorization of restricted antimicrobials was discontinued. Restricted antimicrobials became available for all clinicians to prescribe. To elucidate the consequences of this policy change, we aimed to compare the prescribing of these restricted antimicrobials before and after the removal of prior authorization and to develop a PAF program to mitigate the potential inappropriate prescribing of these antimicrobials.

## Methods

A quasi-experimental design was used to analyze pre- and post-prior authorization intervention data for all Calgary hospitals. Phase 1 included the 24-month period prior to the removal of the prior authorization requirement (May 2020–May 2022). Phase 2 included the 8-month period after the prior authorization requirement was removed (June 2022–January 2023). Phase 3 included a 1-month period where a PAF program was implemented (February 2023). Our objectives were to compare antibiotic utilization data for the restricted antibiotics before and after the removal of the prior authorization requirement and to assess the impact of a PAF program targeting these restricted antimicrobials.

Utilization data was retrospectively collected from a provincial database that tracks inventory movement of the drugs between the pharmacy and hospital units. PAF data was prospectively collected from the electronic medical record. Patients and data were included if they were admitted to a Calgary or Edmonton site and were prescribed meropenem, imipenem-cilastatin, ertapenem, daptomycin, linezolid, and/or cefepime. All Calgary patients prescribed 1 of these agents (excluding cefepime) were eligible for PAF unless they had an infectious diseases consult or were admitted to the intensive care unit.

An interrupted time series analysis was performed to assess antimicrobial utilization. Data points for each study period were analyzed with segmented regression using R software.^3^ Antimicrobial stewardship process measures were analyzed with descriptive statistics. Cefepime utilization throughout the study period was monitored for internal control as a nonequivalent dependent variable. The contemporaneous comparison of Calgary to Edmonton utilization was used for external control. Edmonton does not employ prior authorization and was expected to maintain consistent utilization data throughout the study period. The controls were tested for appropriateness by using the common trend assumption test. Ethics approval was granted by the Health Research Ethics Board at the University of Alberta.

## Results

**Figure 1.**
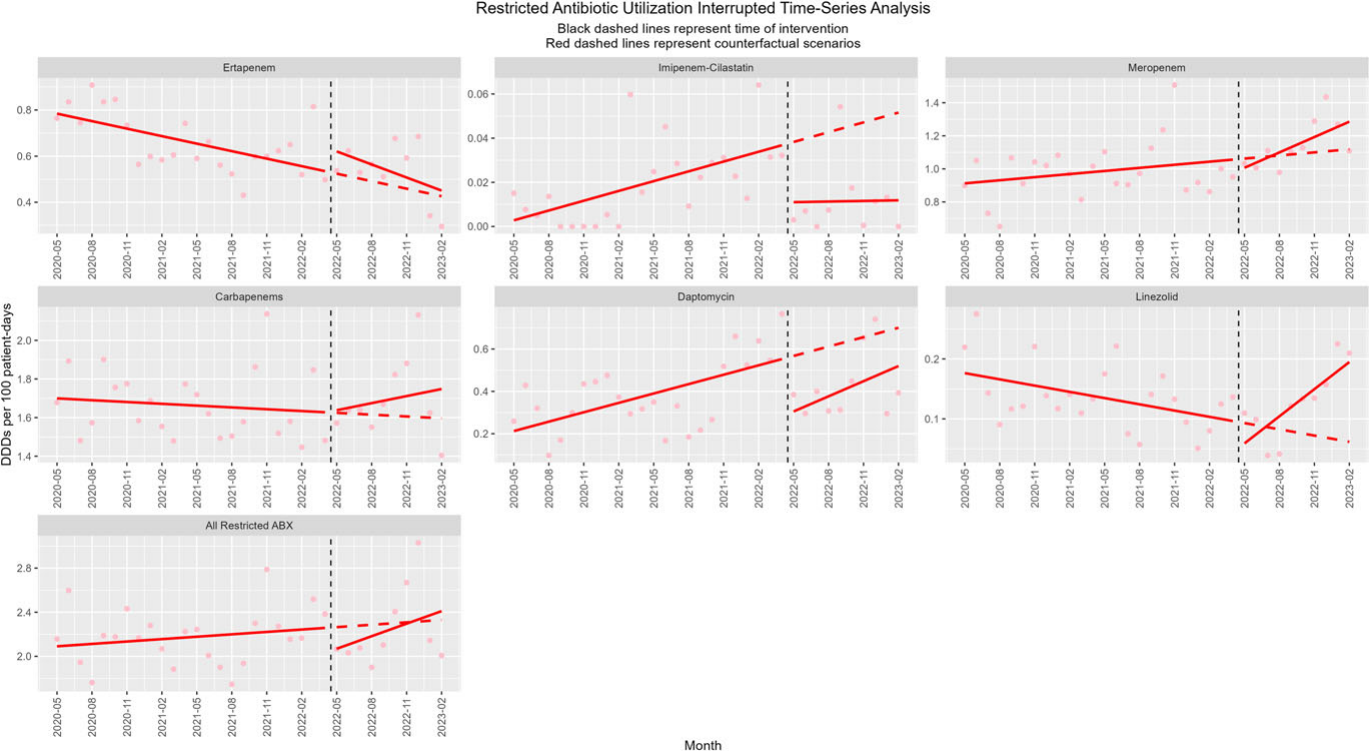
Interrupted time series analysis of antimicrobial utilization before and after formulary restriction removal without controls.

The collective change in restricted antibiotic utilization after the removal of prior authorization was trending toward increased utilization but was not statistically significant (Figure 1). Ertapenem utilization was trending down prior to the study period, and the rate of decrease appeared to accelerate postintervention; however, the effect was not statistically significant. Meropenem also had a statistically nonsignificant change, but the trend favored increased utilization with an acceleration postintervention. The changes in imipenem-cilastatin and daptomycin utilization were statistically significant with a monthly defined daily dose per 100 patient days (DDD/100 PDs) drop of −0.0259 (95% CI, −0.0512–−0.0005) and −0.27 (95% CI, −0.54–−0.006), respectively, which both lost significance with consideration of controls. Linezolid utilization demonstrated a significant increase in trend orientation, rising at 0.019 DDD/100 PDs per month (95% CI, 0.004–0.033), which again became nonsignificant when control variables were analyzed. The collective analysis of carbapenem utilization change surrounding prior authorization removal was nonsignificant, however, demonstrated a trend toward increasing use.

PAF was conducted between January 30 and February 24, 2023, and 133 individual patients were assessed during that period. Thirteen patients (9.8%) were identified by the antimicrobial stewardship pharmacists as requiring intervention for which 19 different recommendations were made, with the most common being to narrow the therapeutic spectrum (47.4%). Stewardship suggestions were accepted 69.2% of the time, which is slightly lower than the site average of 78.4% for a preexisting PAF program with a different focus.

## Discussion

As prior authorization has been demonstrated to mitigate broad-spectrum antibiotic prescribing, the removal of such an intervention could be expected to lead to increased utilization.^1,2^ One study found a significant increase in antimicrobial use (+9.65 days of therapy per 1,000 patient days [DOT/1000 PD] per month; *P* < 0.001), broad-spectrum anti-gram-negative antimicrobial use (+4.80 DOT/1000 PD per month; *P* < 0.001), and longer hospital lengths of stay (*P* = 0.016) when switching from prior authorization to PAF.^4^ Another study found an immediate increase in the utilization of 41.06 DOT/1000 PDs on the ward and 391.04 DOT/1000 PDs in the intensive care unit following the removal of prior authorization.^5^ However, our results suggested a nonsignificant change in utilization in the 9-month period following policy discontinuation, including when PAF was implemented. The brief PAF phase resulted in a few suggested interventions and did not impact utilization. Many of the patients reviewed were already being followed by infectious disease teams, and with only 9.8% of the cohort requiring a stewardship intervention, the change in superfluous prescribing of these drugs was minimal.

Although there were some statistically significant changes detected for a few antibiotics, there were no situations where those changes remained significant after appropriate controls were added to the analyses. As such, the intervention may not have had any statistically significant impact on restricted antibiotic utilization. Further research, exploring the long-term effect of prior authorization removal should be done to further elucidate the implications of broad-spectrum antibiotic utilization.

## References

[1] Barlam TF , Cosgrove SE , Abbo LM , et al. Implementing an antibiotic stewardship program: Guidelines by the Infectious Diseases Society of America and the Society for Healthcare Epidemiology of America. Clin Infect Dis 2016;62:e51–77 27080992 10.1093/cid/ciw118 PMC5006285

[2] Davey P , Marwick CA , Scott CL , et al. Interventions to improve antibiotic prescribing practices for hospital inpatients. *Cochrane Database Syst Rev* 2017;2:CD003543 10.1002/14651858.CD003543.pub4 PMC6464541 28178770

[3] Wagner AK , Soumerai SB , Zhang F , Ross-Degnan D. Segmented regression analysis of interrupted time series studies in medication use research. J Clin Pharm Ther 2002;27:299–309 12174032 10.1046/j.1365-2710.2002.00430.x

[4] Mehta JM. Centers for Disease Control and Prevention Epicenter Program. Comparison of prior authorization and prospective audit with feedback for antimicrobial stewardship. Infect Control Hosp Epidemiol 2014;35:1092–1099 25111916 10.1086/677624 PMC4198070

[5] Jang W , Hwang H , Jo H-U , Cha Y-H , Kim B. Effect of discontinuation of an antimicrobial stewardship programme on the antibiotic usage pattern. Clin Microbiol Infect 2021;27:1860.e1–1860.e5 10.1016/j.cmi.2021.07.019 34325066

